# Mice lacking ASIC2 and βENaC are protected from high-fat-diet-induced metabolic syndrome

**DOI:** 10.3389/fendo.2024.1449344

**Published:** 2024-08-19

**Authors:** Madison Hamby, David E. Stec, Emily Hildebrandt, Donald F. Stec, Heather A. Drummond

**Affiliations:** ^1^ Department of Physiology and Biophysics, University of Mississippi Medical Center, Jackson, MS, United States; ^2^ Department of Chemistry, Vanderbilt University, Nashville, TN, United States

**Keywords:** degenerin, ENaC, ASIC, obesity, high fat diet

## Abstract

**Introduction:**

Degenerin proteins, such as βENaC and ASIC2, have been implicated in cardiovascular function. However, their role in metabolic syndrome have not been studied. To begin to assess this interaction, we evaluated the impact of a high fat diet (HFD) on mice lacking normal levels of ASIC2 (ASIC2^-/-^) and βENaC (βENaC^m/m^).

**Methods:**

Twenty-week-old male and female mice were placed on a 60% HFD for 12 weeks. Body weight was measured weekly, and body composition by non-invasive ECHO MRI and fasting blood glucose were measured at 0, 4, 8 and 12 weeks. A glucose tolerance test was administered after 12 weeks. Differences between ASIC2^-/-^/βENaC^m/m^ and WT groups were compared using independent t-tests or ANOVA where appropriate within each sex. Data are presented as mean ± SEM and ASIC2^-/-^/βENaC^m/m^ vs. WT.

**Results:**

At 20 weeks of age, ASIC2^-/-^/βENaC^m/m^ mice (n=9F/10M) weighed less and gained less weight than WT (n=12F/16M). Total body fat and lean body masses were reduced in female and male ASIC2^-/-^/βENaC^m/m^ mice. Total body fat and lean body masses as % control were identical at the end of 12 weeks. Fasting blood glucoses were lower in female and male ASIC2^-/-^/βENaC^m/m^ vs. WT mice after 12 weeks HFD. The area under the curve for the glucose tolerance test was reduced in female and tended (p=.079) to decrease in male ASIC2^-/-^/βENaC^m/m^. Plasma leptin and insulin were reduced in female and male ASIC2^-/-^/βENaC^m/m^ vs. WT mice. Plasma insulin in female ASIC2^-/-^/βENaC^m/m^ mice remained unchanged throughout the HFD period. Liver and liver fat masses, as well as percent liver fat, were reduced in both female and male ASIC2^-/-^/βENaC^m/m^ mice after HFD. Plasma triglycerides, cholesterol, LDL- and HDL-cholesterols were markedly improved in male and/or female ASIC2^-/-^/βENaC^m/m^ following the HFD.

**Discussion:**

These novel findings suggest that loss of ASIC2 and βENaC offer a significant protection against HFD-induced metabolic syndrome.

## 1 Introduction

Metabolic syndrome is a combination of pathologies that together increase the risk of cardiovascular diseases such as hypertension, stroke, and heart disease (1). The diagnostic criteria for metabolic syndrome include a waist circumference of 40 inches for men and 35 inches for women, reduced HDL of less than 40 mg/dL for men and 50 mg/dL for women, triglycerides of 150 mg/dL or greater, elevated fasting blood glucose of 100 mg/dL or greater, and elevated blood pressure above 135 mmHg systolic or 85 mmHg diastolic (1–3). With the total prevalence of metabolic syndrome rising to over 40% of the general population in recent years (3), it is imperative to understand the mechanisms underlying this disease.

Degenerin proteins are a family of ion channels expressed in a variety of cell types that participate in cardiometabolic control including central and peripheral neurons, smooth muscle cells, immune cells, and epithelial regulation of salt and water transport in the kidney and colon (4–11). While the degenerin channels are expressed in cell types affected by metabolic syndrome and obesity, their role in disease development has never been examined.

The degenerin protein family includes two subfamilies: acid-sensing ion channels (ASICs) and epithelial Na^+^ channels (ENaC). The ASIC subfamily is encoded by five genes: ACCN1, ACCN2, ACCN3, ACCN4, and ACCN5. ASIC channels form homo- and heteromeric inward cation channels that are gated by rapid drops in extracellular pH. ASICs were initially identified in central and peripheral neurons but have also been identified in smooth muscle cells and immune cells (7–10, 12–17). Notably, ASIC2 is expressed in the arcuate nucleus and ventromedial hypothalamus, two areas involved in feeding and satiety (18–20). Our laboratory and others have shown that (1) pressure-induced constriction in small renal arteries and renal afferent arterioles is attenuated, (2) renal autoregulation blood flow is disrupted, and (3) blood pressure is elevated in the ASIC2 global knockout mouse. Taken together, these findings support an important role for ASIC2 in neural and local control of cardiovascular function (12, 21–25).

The epithelial Na^+^ channel (ENaC) subfamily is encoded by four genes (SCNN1a, SCNN1b, SCNN1g, and SCNN1d) and forms heterotrimeric, non-voltage gated, amiloride-sensitive sodium channels predominantly expressed in renal, colon, and lung epithelia and maintains sodium and water homeostasis (6, 26). ENaC subunits have also been identified in sensory neurons, endothelial cells, choroid plexus, select central nervous system neurons, immune cells, small intestinal enterocytes, and vascular smooth muscle cells. The classical ENaC channel is a heterotrimer formed by α, β, and γ subunits. The delta subunit is specific to humans and can form a channel with βγENaC (26–30). Our laboratory has focused on the importance of βENaC in the cardiovascular system due to its expression in sensory neurons and vascular smooth muscle cells. Gain of function mutations associated with βENaC include Liddle’s syndrome, characterized by severe hypertension due to excess salt and water retention in the distal nephron (31). Loss of function mutations are associated with autosomal recessive pseudohyperaldosteronism type I, characterized by renal salt and water wasting (26, 32). Due to the importance of ENaC in salt and water homeostasis, few animal models of gain or loss of function are available. Because of this, our laboratory uses a mouse hypomorphic βENaC model (βENaC^m/m^) developed by the Hummler laboratory (32). In addition to salt wasting under severe dietary Na^+^ restriction, our laboratory has shown that βENaC hypomorphic mice also have attenuated (1) pressure-induced constriction in small renal arteries and renal afferent arterioles, (2) autoregulation of renal blood flow, and (3) arterial baroreflex control of blood pressure, leading to elevated arterial blood pressure and enhanced blood pressure variability (25, 27–29, 33).

What evidence suggests that ASIC2 or βENaC might contribute to the metabolic syndrome and obesity? As noted above, ASIC2 and βENaC are expressed in many cell types involved in the pathology including adipocytes, hepatocytes, central neurons, endothelial cells, vascular smooth muscle cells, and immune cells (4–7, 13, 20, 22, 23, 26, 27, 29, 30, 34–39). Along with its involvement in neural and local vascular control of cardiovascular function, βENaC may also be a target for the treatment of systemic metabolic dysfunction (6, 27, 29, 36, 37). It is known that ENaCs are regulated by insulin, and sodium influx into the cell allows for the activation of other ion channels and signaling cascades (6, 37). Loss of function of at least two degenerin proteins, ASIC2 or βENaC, leads to disrupted cardiovascular function including arterial baroreceptor, myogenic regulation of cerebral and renal blood flow, and hypertension (22, 24, 27). More compelling, ASIC2, and βENaC to a lesser extent, is expressed throughout the hypothalamus, specifically in hypothalamic and nucleus tractus solitarius areas important in the homeostasis of feeding, satiety, and blood pressure regulation (15, 16, 18–20, 40, 41). The potential importance of degenerin proteins ASIC2/βENaC in the development of metabolic syndrome has not been previously explored. In the current investigation, we provide evidence that male and female mice lacking normal levels of ASIC2 and βENaC are protected from metabolic dysfunction and hepatic steatosis associated with a high-fat diet.

## 2 Materials and methods

### 2.1 Experimental design and body composition

All animal works were done under the Association for Assessment and Accreditation of Laboratory Animal Care (AAALAC) and the University of Mississippi Medical Center (UMMC) Institutional Animal Care and Use Committee (IACUC). The model was generated by crossing ASIC2 global knockout mice (ASIC2^-/-^; RRID: IMSR_JAX:013126) mice onto the hypomorphic βENaC strain (βENaC^m/m^, RRID: IMSR_EM:04574) as described previously (25). The animals were maintained as homozygous mating pairs. The mice were maintained under a 12-h light/12-h dark light cycle and permitted food and water *ad libitum*. Male and female mice were used in this study. There were no exclusion criteria. After analysis, genotypes were confirmed in all animals using liver DNA. All protocols were approved by the Institutional Animal Care and Use Committee at UMMC.

At 20 weeks of age, male and female wild-type and ASIC2^-/-^/βENaC^m/m^ mice were placed on a high-fat diet (HFD; 60% kcal from fat, 21% kcal from carbohydrate, Envigo, cat. #TD.0644) or remained on a normal chow (NFD; 14% kcal fat, 54% kcal carbohydrate, Envigo, cat. #TD.8604) for 12 weeks (*n* = 6–16/group). During this period, the mice were housed in home cages. Body mass was obtained weekly. Body composition was assessed at weeks 0, 4, 8, and 12 of NFD or HFD using Echo MRI (4-in-1 EchoMRI-900TM, Echo Medical System, Houston, TX, USA). Additionally, a 6-h fasting blood sample was collected via retro-orbital eye bleed for analysis of glucose, insulin, and leptin. At 12 weeks, a glucose tolerance test was performed on a group of HFD animals. Upon completion of the study, the mice were fasted and their organs were collected, weighed, then snap-frozen in liquid nitrogen, and stored at -70°C, except the livers.

### 2.2 Glucose tolerance test

For the glucose tolerance test, the mice were injected with 1 mg glucose/kg body mass intra-peritoneally, and blood samples were collected at 15, 30, 45, 60, and 90 min after injection. Blood glucose was assessed using an Accu-check glucose meter.

### 2.3 Plasma assays

Plasma leptin (R&D Systems, cat. #MOB00B) and insulin (Crystal Chem, cat. #90080) were assessed by ELISA through the Analytical and Assay Core in the Department of Physiology and Biophysics at UMMC. The Analytical and Assay Core uses a standardized approach and internal standards to provide a repeatable and reliable assessment. Plasma lipids were assessed through 1H nuclear magnetic resonance (NMR) spectroscopy using the Bruker Biospin IVDr platform in the Center for Structural Biology NMR Core Facility at Vanderbilt University as previously described (42–44).

### 2.4 Anthropometric measurements

To obtain body length data, we measured the distance between the tip of the nose to the base of the tail using a ruler (mm). To obtain tibial length data, we measured the distance from the ankle to the knee using a ruler (mm).

### 2.5 Liver fat accumulation

Livers were assessed for fat content using EchoMRI and then fractioned for fixation in 4% paraformaldehyde for Oil Red O staining in cryosections through the Histology Core in the Department of Physiology and Biophysics at UMMC (44, 45). The remaining liver samples were stored at -70°C.

### 2.6 Liver macrophage localization

Liver cryosections (20 µm) were stained with rat anti-F4/80 (Biorad, cat. #MCA497, RRID AB_2098196) to label macrophages. Tissue sections were rinsed with PBS, permeabilized with 0.1% Triton X-100 (Thermo, cat. #28314), blocked with 5% normal donkey serum (NDS, Jackson Immuno, cat. #017-000-121), incubated with primary antibody (1:25–1:100) overnight in 5% NDS, rinsed again, incubated with donkey anti-rat Cy3 (1:250, Jackson Immuno, cat. #712-165-153, RRID AB_2340667) for 1 h, then rinsed, and cover-slipped. Liver macrophage localization was visualized using a Leica SP8 laser scanning confocal microscope. The samples were imaged under identical conditions using a ×63 objective and prepared identically for publication in Adobe Photoshop. Negative control samples lacking primary antibody were run in parallel and used to establish baseline fluorescence signal. Violin plots representing the quantification of liver macrophages are representative of 15 fields of view from *n* = 2 animals per group.

### 2.7 Quantification and statistical analysis

Data were preliminarily analyzed in Excel and then transferred to GraphPad Prism for statistical analysis. Data are presented as mean ± standard error mean, except for immunofluorescence quantitation data which are presented as violin plots with median and quartiles. Data were analyzed using multiple approaches including repeated-measures ANOVA, two-way, or one-way ANOVA where appropriate, followed by Holm’s Sidak *post hoc* analysis. Male and female groups were analyzed separately due to unequal sample sizes precluding a three-way ANOVA analysis in Prism. Lipid profile data were analyzed using dependent, two-tailed *t*-tests with the Bonferroni correction. Figures and figure legends identify sample sizes, specific statistical and *post hoc* analyses, and significance notations or *p*-values.

## 3 Results

### 3.1 Time course for body mass gain in WT and ASIC2^-/-^/βENaC^m/m^ mice gain in response to NFD and HFD

**Figure 1:**
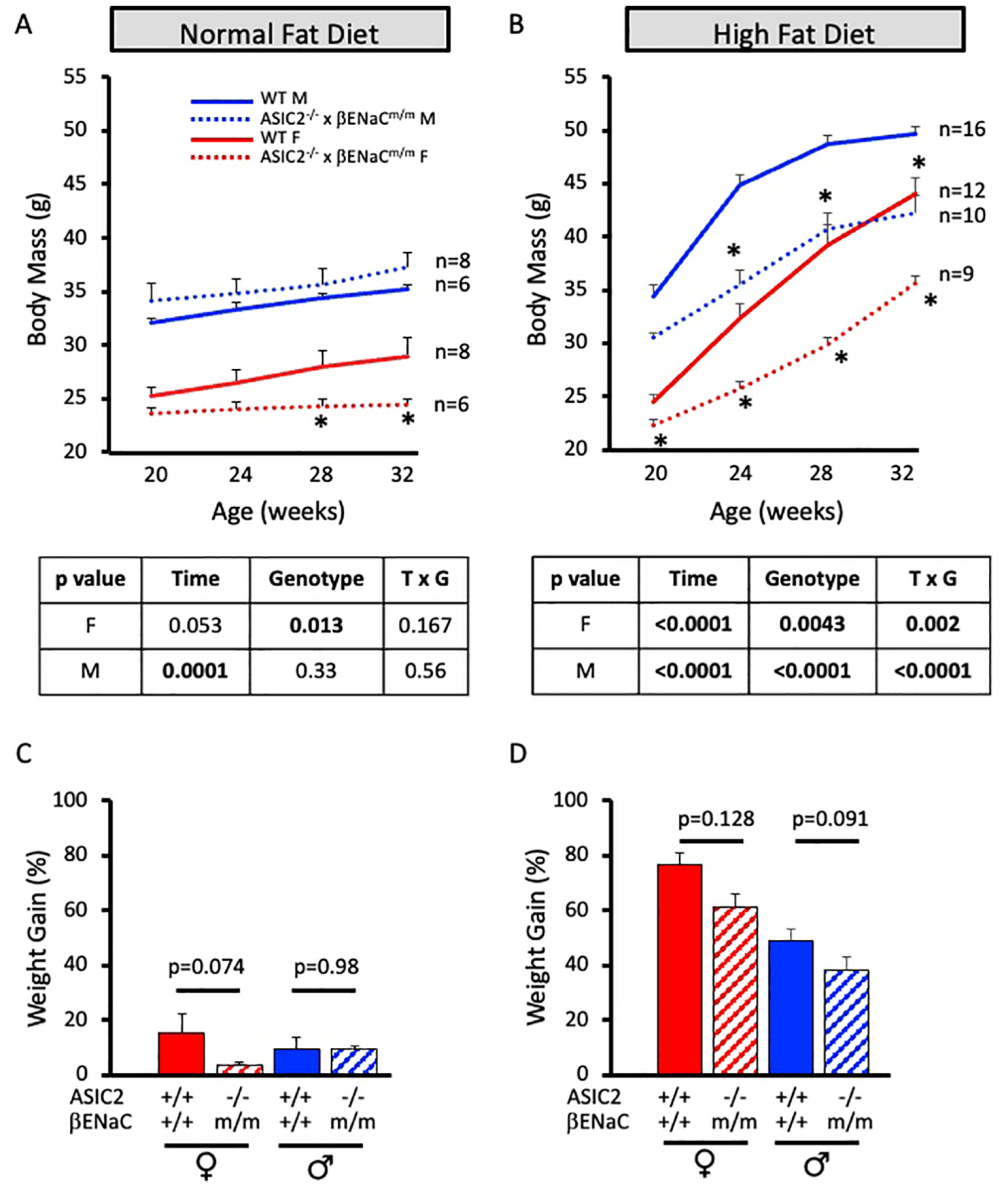
ASIC2^-/-^/βENaC^m/m^ mice gain less body mass with 60% high-fat diet (HFD) from 20 weeks of age to 32 weeks of age. **(A)** Body mass change in normal-fat diet (NFD) ASIC2^-/-^/βENaC^m/m^ and wild-type (WT) control animals. **(B)** Body mass change in HFD-fed ASIC2^-/-^/βENaC^m/m^ and WT control animals. **(C, D)** Weight gain over the 12-week period in NFD- and HFD-fed groups. Red lines/columns represent female individuals; blue lines/bars represent male individuals. Solid lines and columns represent the WT group. Dashed lines and hatched columns represent ASIC2^-/-^/βENaC^m/m^. Sample sizes are shown in **(A, B)**. Data in **(A, B)** were analyzed using repeated-measures ANOVA within sex, followed by Holm’s Sidak *post hoc* analysis. The asterisk denotes being significantly different from WT at a given time point at *p <*0.05. Data in **(C, D)** were analyzed using independent *t*-tests within sex; *p*-values are provided on the graph.

To determine the role of ASIC2/βENaC on HFD-induced body mass gain, we measured the body weight of ASIC2^-/-^/βENaC^m/m^ and WT mice every 4 weeks while on NFD (**Figure 1A**) and HFD (**Figure 1B**). As shown in **Figure 1A**, ASIC2^-/-^/βENaC^m/m^ male mice on the NFD gain mass similar to WT controls, while ASIC2^-/-^/βENaC^m/m^ female mice gain less body mass compared to WT controls. When fed a HFD, both male and female ASIC2^-/-^/βENaC^m/m^ mice have a significantly lower body mass at 4, 8, and 12 weeks compared to WT mice (**Figure 1B**). *P*-values for the two-way ANOVA are provided in the tables immediately below **Figures 1A, B**. Percent weight gain values for NFD and HFD groups over the 12-week period are shown in **Figures 1C, D**, respectively. The percent weight gain tended to be lower in ASIC2^-/-^/βENaC^m/m^ female mice vs. WT mice on NFD, while ASIC2^-/-^/βENaC^m/m^ male mice gained a similar relative amount of body mass over 12 weeks on NFD (**Figure 1C**). The percent body mass gain in male and female ASIC2^-/-^/βENaC^m/m^ groups on HFD trends toward lower body mass gains following the 12-week HFD compared to WT mice (**Figure 1D**).

### 3.2 Body composition is similar, but not identical, in ASIC2^-/-^/βENaC^m/m^ and WT mice on NFD and HFD

**Figure 2:**
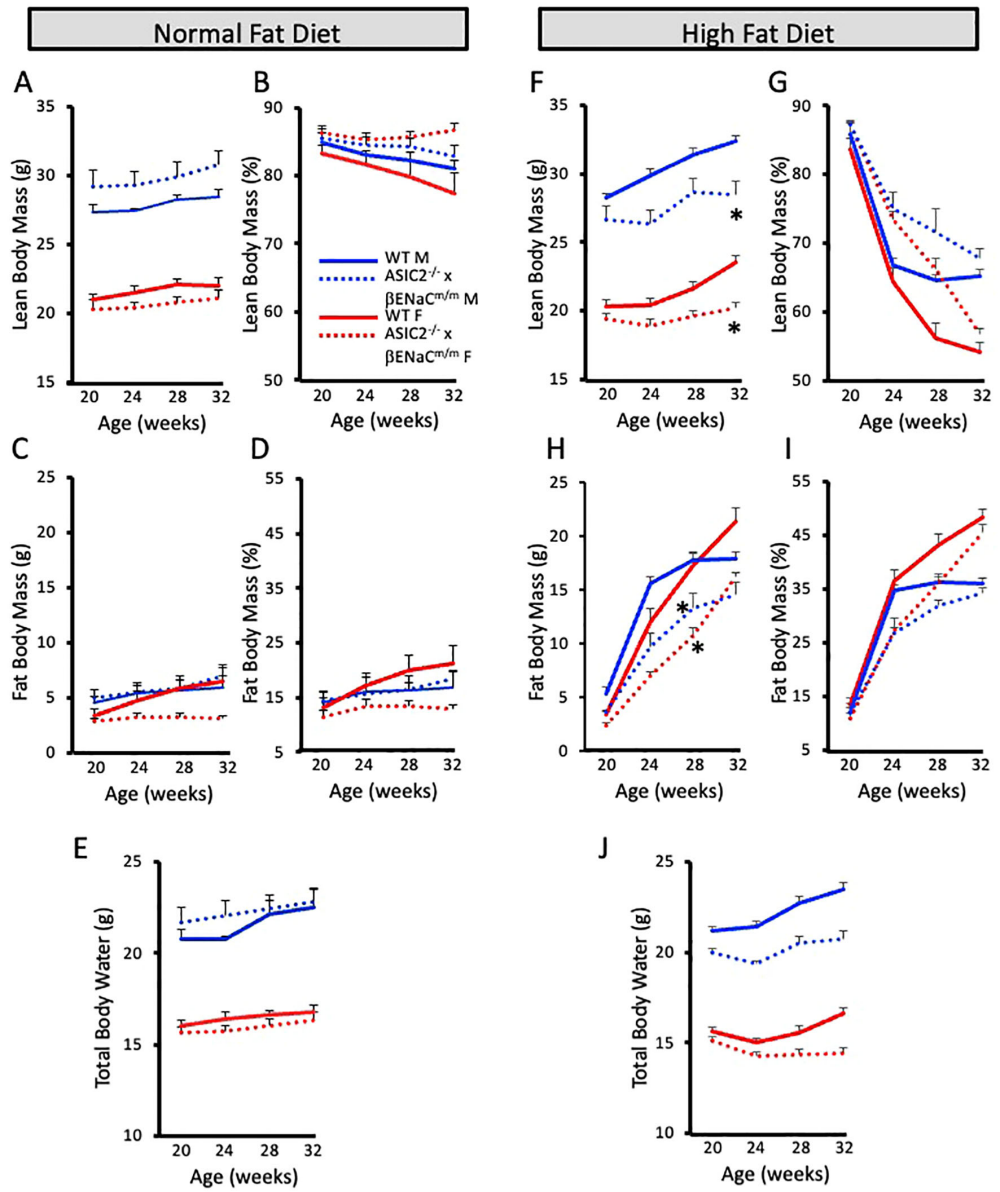
Body composition is similar, but not identical, in ASIC2^-/-^/βENaC^m/m^ and wild-type (WT) mice on a 60% high-fat diet (HFD) from 20 weeks of age to 32 weeks of age. **(A, B)** Absolute and normalized lean body mass in normal-fat diet (NFD) mice. **(C, D)** Absolute and normalized fat body mass in NFD mice. **(E)** Total body water content in NFD mice. **(F, G)** Absolute and normalized lean body mass in HFD mice. **(H, I)** Absolute and normalized fat body mass in HFD mice. **(J)** Total body water content in HFD mice. Red lines/bars represent female individuals; blue lines/bars represent male individuals. Solid lines represent the WT group, and dashed lines represent ASIC2^-/-^/βENaC^m/m^. * Significantly different from control at respective time point.

We used non-invasive ECHO MRI to determine the body composition changes in NFD and HFD animals. We measured lean mass, fat mass, and total body water of ASIC2^-/-^/βENaC^m/m^ and WT mice every 4 weeks while on diet. On the NFD, there were no significant differences in absolute and normalized lean or fat body masses or total body water (**Figures 2A–E**). After 12 weeks of HFD, lean and fat body masses were significantly lower in female and male ASIC2^-/-^/βENaC^m/m^ mice at 4, 8, and 12 weeks compared to WT mice (**Figures 2F, H**); however, normalized lean and fat body masses were similar at the end of the 12-week HFD period (**Figures 2G, I**). Total body water content was lower in male and female ASIC2^-/-^/βENaC^m/m^ mice on HFD but not the NFD group, which was likely associated with their lower absolute lean body mass (**Figure 2J**).

### 3.3 Fasting blood glucose is lower in female and male ASIC2^-/-^/βENaC^m/m^ mice fed HFD

**Figure 3:**
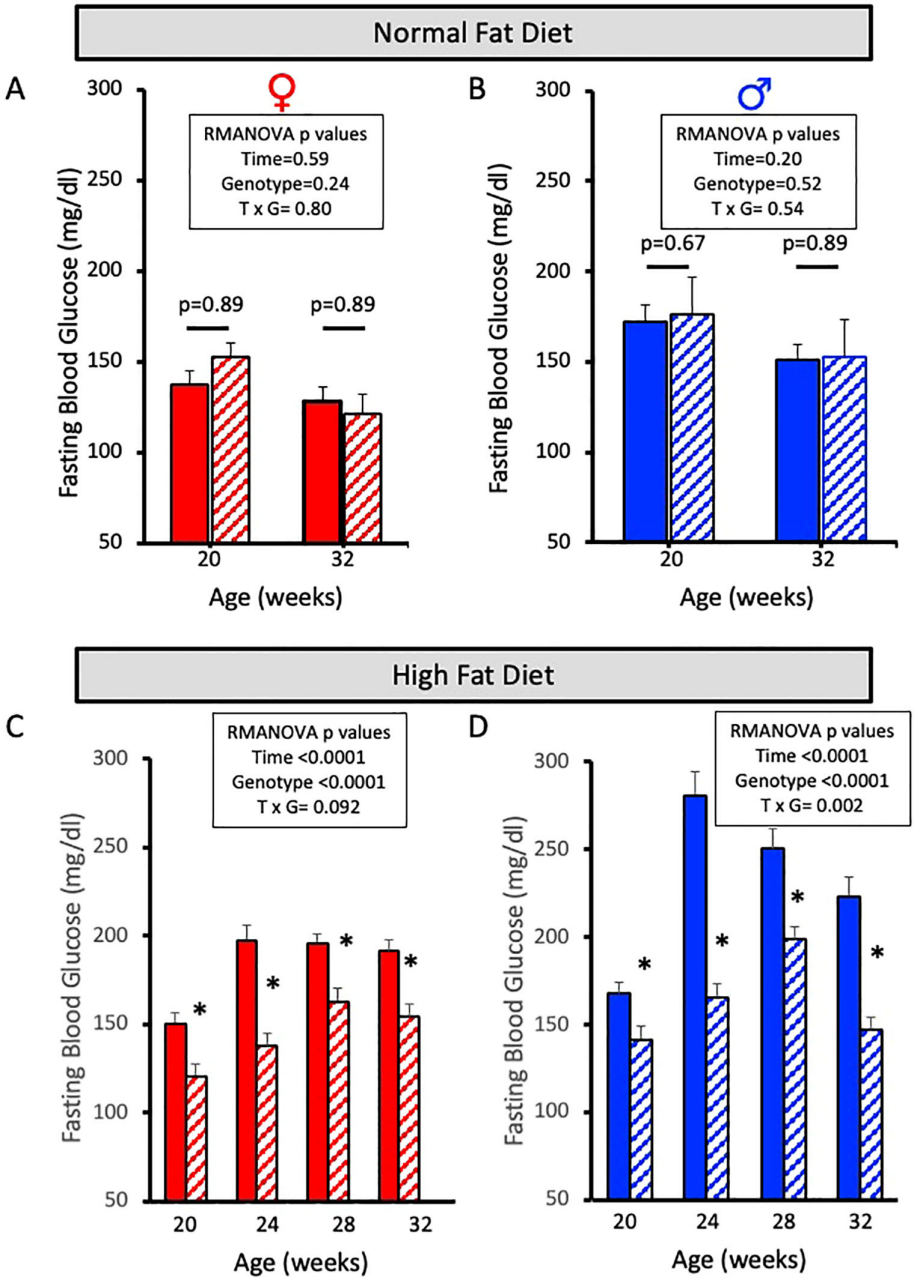
Fasting blood glucose is lower in female and male ASIC2^-/-^/βENaC^m/m^ mice fed a high-fat diet (HFD) for 12 weeks. **(A, B)** Fasting blood glucose in normal-fat diet (NFD) female and male ASIC2^-/-^/βENaC^m/m^ and wild-type (WT) mice at 20 and 32 weeks of age. **(C, D)** Fasting blood glucose in HFD female and male ASIC2^-/-^/βENaC^m/m^ and WT mice at 20 and 32 weeks of age. Red columns represent female individuals; blue columns represent male individuals. Solid columns represent the WT group, and hatched columns represent the ASIC2^-/-^/βENaC^m/m^ group. Data in all panels were analyzed using repeated-measures ANOVA (*p*-values shown on the graph), followed by Holm’s Sidak *post hoc* test. *Post hoc P*-values shown in A/B. The asterisk denotes being significantly different than WT at a given time point at *p <*0.05.

Fasting blood glucose levels were obtained at 0, 4, 8, and 12 weeks for the NFD (**Figures 3A, B**) and HFD (**Figures 3C, D**) groups via retro-orbital eye bleed using an Accu-check glucometer. Female and male ASIC2^-/-^/βENaC^m/m^ and WT mice fed a NFD for 12 weeks have no significant changes in fasting blood glucose levels (**Figures 3A, B**). However, both female and male ASIC2^-/-^/βENaC^m/m^ mice fed a HFD have significantly reduced fasting blood glucose levels at each 4-week timepoint tested compared to their WT counterparts (**Figures 3C, D**).

### 3.4 Glucose tolerance is improved in female ASIC2^-/-^/βENaC^m/m^ mice fed HFD

**Figure 4:**
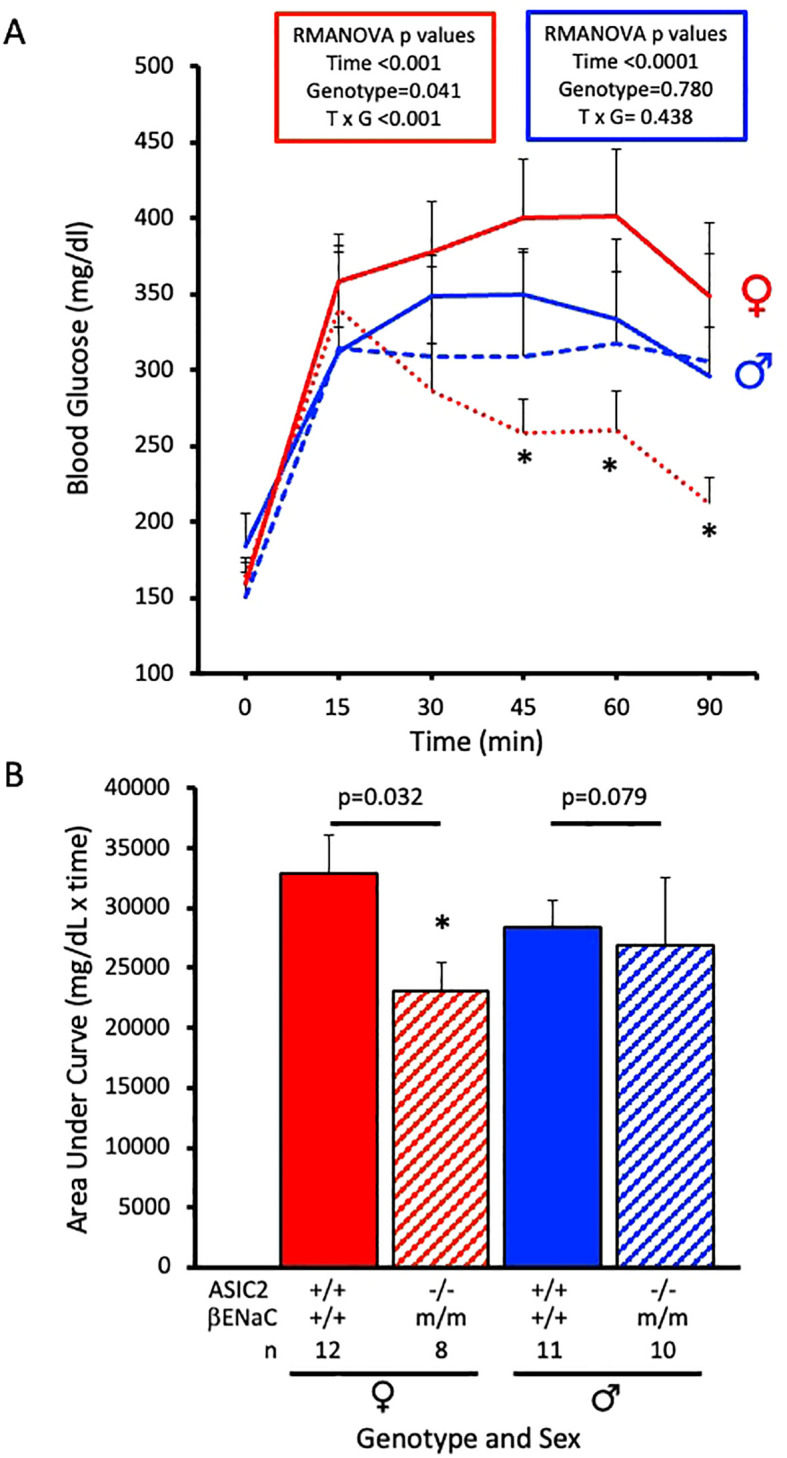
Glucose tolerance is improved in ASIC2^-/-^/βENaC^m/m^ female mice fed a high-fat diet (HFD) for 20 weeks. **(A)** Changes in plasma glucose following a bolus IP injection of glucose in female and male ASIC2^-/-^/βENaC^m/m^ and wild-type (WT) mice. The data in **(A)** were analyzed using repeated-measures analysis of variance for each sex (*p*-values shown on the graph), followed by Holm’s Sidak *post hoc* test. The asterisk denotes being significantly different from WT at a respective time point, *p* < 0.05. **(B)** Area under the curve for HFD female and male ASIC2^-/-^/βENaC^m/m^ and WT mice. Data in **(B)** were analyzed using two-tailed, independent *t*-tests with *p*-values shown on the graph. Red lines/columns represent female individuals; blue lines/columns represent male individuals. Solid lines/columns represent the WT group; dashed lines/hatched columns represent the ASIC2^-/-^/βENaC^m/m^ group.

Glucose tolerance tests were performed after a 12-week HFD. Changes in plasma glucose were measured at 0, 15, 30, 45, 60, and 90 min post-glucose bolus using retro-orbital eye bleed samples. Male ASIC2^-/-^/βENaC^m/m^ and WT mice exhibited similar increases in blood glucose response time course, while ASIC2^-/-^/βENaC^m/m^ female mice showed a rapid decline in plasma glucose compared to WT mice (**Figure 4A**). The areas under the curve for male ASIC2^-/-^/βENaC^m/m^ trended lower, and the WT mice were not significantly different. The female ASIC2^-/-^/βENaC^m/m^ mice had a significantly reduced area under the curve compared to WT animals (**Figure 4B**).

### 3.5 ASIC2^-/-^/βENaC^m/m^ mice are protected from HFD-induced increases in plasma insulin and leptin

**Figure 5:**
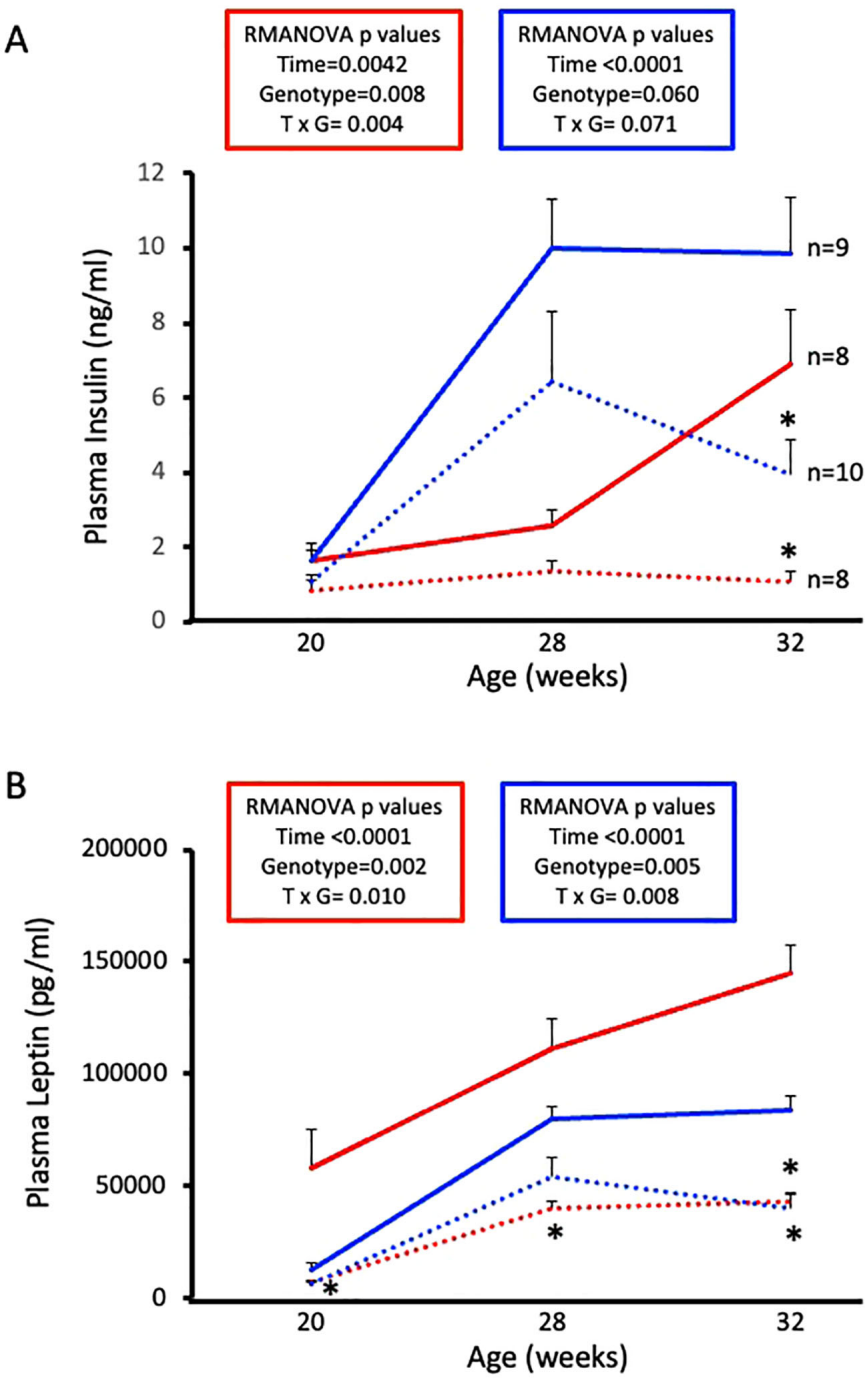
High-fat diet (HFD)-induced increases in plasma insulin and leptin are attenuated in female and male ASIC2^-/-^/βENaC^m/m^ mice. **(A)** Plasma insulin of HFD female and male ASIC2^-/-^/βENaC^m/m^ and wild-type (WT) mice. **(B)** Plasma leptin of HFD female and male ASIC2^-/-^/βENaC^m/m^ and WT mice. Data were analyzed using repeated-measures analysis of variance followed by Holm’s Sidak *post hoc* test. *P*-values for main and interacting effects of time and genotype are provided on the graphs. The asterisk denotes being significantly different from WT control at a respective time point. Red lines represent female individuals; blue lines represent male individuals. Solid lines represent the WT group; dashed lines represent the ASIC2^-/-^/βENaC^m/m^ group.

Next, we wanted to examine circulating hormones that play a role in glucose homeostasis. Plasma insulin and leptin levels were determined by ELISA using samples collected at diet initiation day, 8 weeks on diet, and 12 weeks on diet. Male and female WT mice exhibit expected HFD-induced increases in plasma insulin and leptin (**Figures 5A, B**, solid lines). Male and female ASIC2^-/-^/βENaC^m/m^ mice show reduced circulating insulin and leptin at 12 weeks on HFD (**Figures 5A, B**, dotted lines). Notably, plasma insulin was stable in female ASIC2^-/-^/βENaC^m/m^ mice throughout the study.

### 3.6 Organ and fat depot masses: Liver mass and intra-abdominal fat mass are lower in female and male ASIC2^-/-^/βENaC^m/m^ mice fed HFD

Anthropomorphic measurements were performed before (20 weeks old) and after (32 weeks old) 12 weeks on HFD (**Table 1**). Female and male ASIC2^-/-^/βENaC^m/m^ and WT mice fed a NFD for 12 weeks have no significant differences in body mass, body length, or tibial length. However, ASIC2^-/-^/βENaC^m/m^ mice fed a HFD had lower body masses, body length (female mice only), and tibial length. Select differences in organ masses were also noted in ASIC2^-/-^/βENaC^m/m^ animals—for example, in both female and male ASIC2^-/-^/βENaC^m/m^ mice fed a NFD, the heart, kidney, liver, pelvic fat, and visceral fat mass metrics are similar, with a few sporadic exceptions, with normalization. In contrast, ASIC2^-/-^/βENaC^m/m^ mice fed a HFD have significantly reduced liver mass, intra-abdominal fat, and visceral fat masses following 12 weeks of HFD compared to WT. Interestingly, normalized heart mass is also lower in female ASIC2^-/-^/βENaC^m/m^ mice after HFD compared to WT controls. Similar to our current investigation, we previously found normalized heart mass in 13-week-old, NFD-fed male ASIC2^-/-^/βENaC^m/m^ mice, which was identical to control animals despite an 8-mmHg increase in mean arterial blood pressure (25). We did not measure blood pressure in the current investigation.

**Table 1:** Morphometrics of ASIC2^+/+^/βENaC^+/+^ and ASIC2^-/-^/βENaC^m/m^ mice before (20 weeks old) and after (32 weeks old) a 12-week high-fat diet.

Diet	Normal-fat diet				High-fat diet			
Sex	Female		Male		Female		Male	
Genotype ASIC2	+/+	-/-	+/+	-/-	+/+	-/-	+/+	-/-
βENaC	+/+	m/m	+/+	m/m	+/+	m/m	+/+	m/m
Sample size	8	6	5	8	10	7	16	10
Age (weeks)	32.7 ± 0.3	32.6 ± 0.1	32.7 ± 0.2	32.6 ± 0.4	32.2 ± 0.1	32.5 ± 0.1	32.1 ± 0.1	32.5 ± 0.1
Body mass (g)	27.0 ± 1.7	23.0 ± 0.4	33.5 ± 0.6	35.5 ± 1.8	44.2 ± 1.9	33.7 ± 0.7*	47.9 ± 0.7	39.6 ± 1.5*
Body length (cm)	9.7 ± 0.2	9.3 ± 0.1	9.7 ± 0.9	10.0 ± 0.2	10.4 ± 0.1	9.9 ± 0.1*	10.6 ± 0.1	10.6 ± 0.1
Tibial length (cm)	2.0 ± 0.07	2.1 ± 0.1	2.1 ± 0.07	2.2 ± 0.1	1.96 ± 0.02	2.15 ± 0.03*	2.23 ± 0.03	2.07 ± 0.01*
Spleen (mg)	80 ± 5	121 ± 28	111 ± 14	103 ± 6	133 ± 10	107 ± 14	122 ± 7	175 ± 36
Heart mass (mg)	120 ± 6	130 ± 8	139 ± 17	189 ± 10*	167 ± 6	115 ± 2*	183 ± 4	170 ± 6
HM/BM (mg/g)	4.5 ± 0.3	5.6 ± 0.3*	4.2 ± 0.5	5.3 ± 0.4	3.8 ± 0.1	3.4 ± 0.8*	3.8 ± 0.1	4.3 ± 0.1*
HM/BL (mg/cm)	12.3 ± 0.7	13.9 ± 0.8	14.3 ± 1.7	18.7 ± 1.3	16.1 ± 0.6	11.6 ± 0.4*	17.3 ± 0.4	16.1 ± 0.5*
HM/TL (mg/cm)	60.7 ± 4.8	62.0 ± 2.6	67.8 ± 8.5	86.3 ± 7.2	77.7 ± 3.3	58.9 ± 0.6*	82.4 ± 2.4	81.9 ± 2.6
Kidney mass (mg)	263 ± 16	257 ± 9	364 ± 44	425 ± 29	298 ± 14	249 ± 10*	379 ± 14	371 ± 18
KM/BM (mg/g)	9.8 ± 0.3	11.2 ± 0.3	10.8 ± 1.3	13.4 ± 1.7	6.8 ± 0.2	7.4 ± 0.2*	7.9 ± 0.2	9.4 ± 0.3*
KM/BL (mg/cm)	27.3 ± 1.3	27.6 ± 1.0	37.2 ± 4.2	46.8 ± 5.6	28.8 ± 1.3	25.7 ± 1.1	35.9 ± 1.3	35.1 ± 1.6
KM/TL (mg/cm)	134.2 ± 10.7	124.0 ± 3.8	176.0 ± 18.1	219.1 ± 30.0	138.8 ± 6.5	127.0 ± 4.9	170.7 ± 6.2	178.6 ± 7.8
Liver mass (mg)	1,019 ± 57	1,008 ± 38	1,456 ± 31	1,415 ± 75	1,966 ± 326	940 ± 43*	2,560 ± 135	1,692 ± 153*
LM/BM (mg/g)	38 ± 2	44 ± 1*	43 ± 1	40 ± 2	43 ± 6	28 ± 1*	53 ± 2	42 ± 3*
LM/BL (mg/cm)	105 ± 5	108 ± 4	149 ± 3	141 ± 7	189 ± 31	95 ± 4*	243 ± 13	160 ± 14*
LM/TL (mg/cm)	515 ± 37	484 ± 16	708 ± 17	656 ± 42	909 ± 146	481 ± 22*	1151 ± 61	815 ± 72*
Intra-abdominal fat (g)	1.5 ± 0.4	0.7 ± 0.1	1.6 ± 0.3	2.1 ± 0.2	6.3 ± 0.5	4.3 ± 0.2*	4.1 ± 0.3	3.2 ± 0.1*
Pelvic fat (g)	1.2 ± 0.4	0.5 ± 0.1	0.9 ± 0.3	1.2 ± 0.1	3.2 ± 0.2	2.6 ± 0.2	1.8 ± 0.1	1.4 ± 0.1*
Visceral fat (g)	0.3 ± 0.1	0.2 ± 0.1	0.7 ± 0.2	0.9 ± 0.1	3.1 ± 0.3	1.8 ± 0.1*	2.3 ± 0.2	1.8 ± 0.1*

*p < 0.05 vs. ASIC2^+/+^/βENaC^+/+^ control (within gender and diet), independent two-tailed t-test.

BM, body mass; BL, body length; TL, tibial length; HM, heart mass; KM, kidney mass; LM, liver mass.

### 3.7 ASIC2^-/-^/βENaC^m/m^ mice fed HFD are protected from liver steatosis and macrophage accumulation

**Figure 6:**
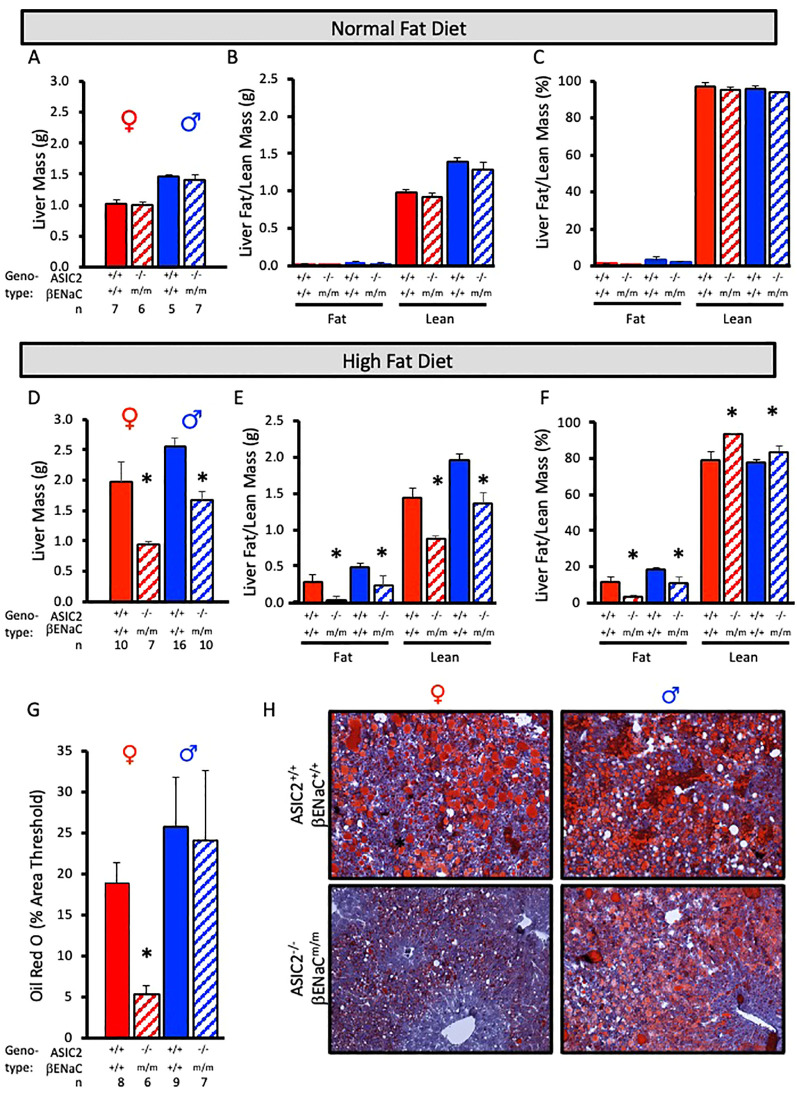
Liver mass and fat mass are reduced in ASIC2^-/-^/βENaC^m/m^ fed a high-fat diet (HFD) for 20 weeks. **(A-C)** Total liver, liver lean, and liver fat masses in normal-fat diet (NFD) ASIC2^-/-^/βENaC^m/m^ and wild-type (WT) mice. **(D–F)** Total liver, liver lean, and liver fat masses in HFD ASIC2^-/-^/βENaC^m/m^ vs. WT mice. **(G)** Group data showing quantitative Oil Red O staining as percent of image area. **(H)** Representative images of Oil Red O staining in WT (top row) vs. ASIC2^-/-^/βENaC^m/m^ (bottom row) NFD mice. Female individuals (left column) and male individuals (right column). All data were analyzed using a two-tailed, independent *t*-test for each sex. The asterisk denotes being significantly different from the WT group, *p* < 0.05. Solid columns represent the WT group, and hatched columns represent the ASIC2^-/-^/βENaC^m/m^ group.

To examine fat accumulation in the liver, we examined liver weight and liver fat and lean masses using ECHO MRI and Oil Red O staining of liver tissue. Liver mass, liver lean mass, and liver fat mass were identical in ASIC2^-/-^/βENaC^m/m^ and WT mice fed a NFD (**Figures 6A–C**). In contrast, male and female ASIC2^-/-^/βENaC^m/m^ mice on HFD had a lower liver mass (**Figure 6D**) as well as lower absolute lean and fat masses (**Figure 6E**) and percent liver fat (**Figure 6F**) compared to their WT counterparts (**Figures 6D–F**). As a secondary approach to assess liver fat content, we used Oil Red O staining of frozen liver sections. The quantitative group data and representative images in **Figures 6G, H** show the lower liver fat content in female, but not male, ASIC2^-/-^/βENaC^m/m^ mice. Representative quantitation revealed a reduced staining percent of image area in female ASIC2^-/-^/βENaC^m/m^ mice compared to female WT mice (**Figures 6G, H**). Our model uses lard as the primary source of fat in the HFD. Lard is a common source of animal fat and is widely used in animal studies for this reason (42–45).

**Figure 7:**
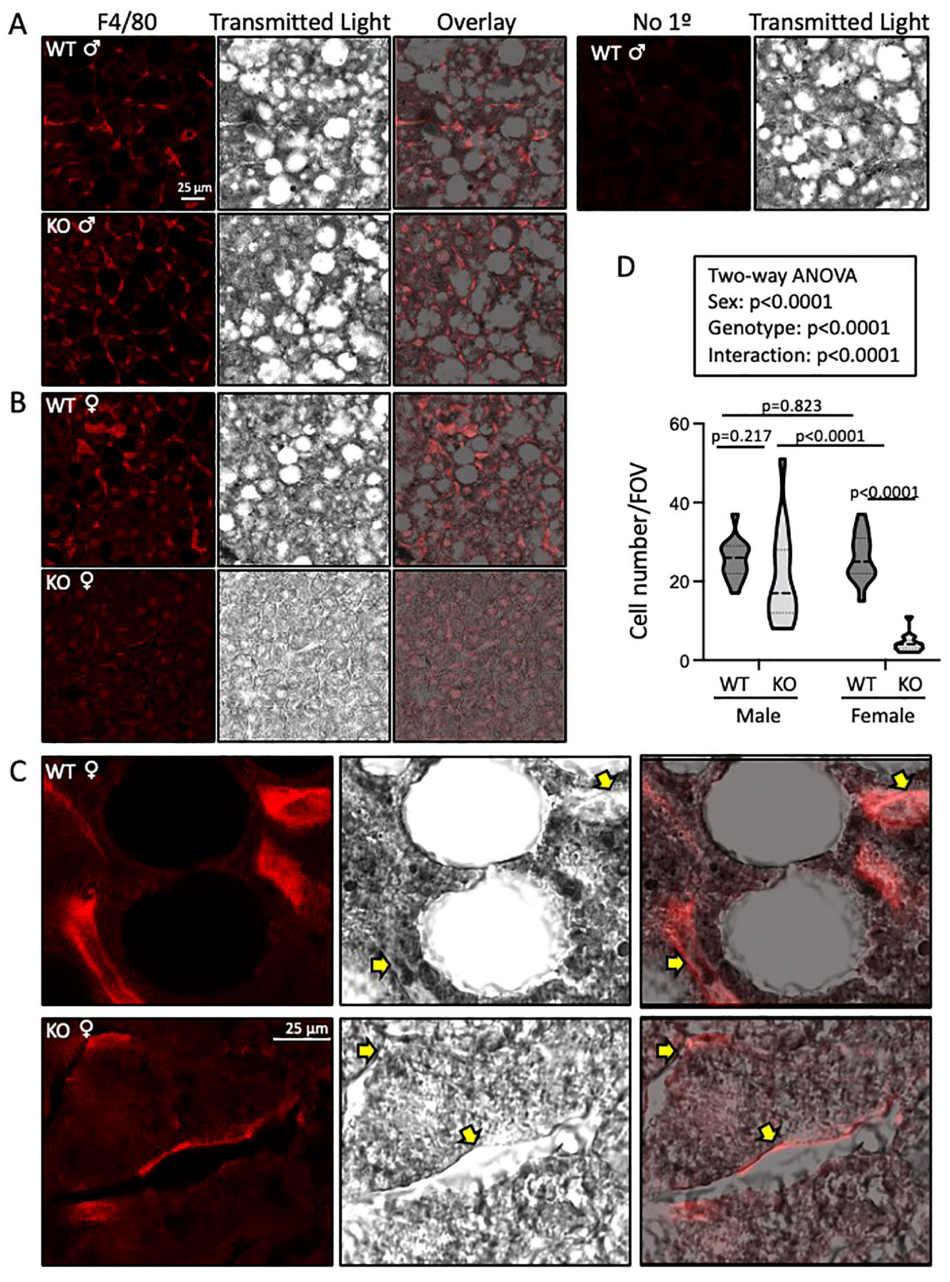
Liver macrophage localization (F4/80 labeling) is reduced in ASIC2^-/-^/βENaC^m/m^ high-fat diet (HFD) female mice. **(A-C)** Representative images of F4/80-labeled macrophages (red, left), transmitted light (middle, gray scale), and an overlay (right). **(A, B)** Images were obtained at ×63. Macrophage number and F4/80 intensity were observed in wild-type (WT; top panels) *versus* ASIC2^-/-^/βENaC^m/m^ (bottom panels) liver sections. A negative control sample lacking the F4/80 antibody is shown in the top row, right-hand side. **(C)** High magnification (×63 objective plus ×3 optical zoom), images of F4/80-labeled cells in WT and ASIC2^-/-^/βENaC^m/m^ female mice’ livers. **(D)** The truncated violin plot represents group data showing the number of F4/80-positive cells (15 fields of view at ×63 from one section from two different animals/group). Data, presented as median and quartiles, were analyzed using two-way analysis of variance, followed by Holm’s Sidak *post hoc* test. *P*-values are provided on graph.

We used immunofluorescence to assess macrophage infiltration using F4/80 labeling in frozen liver sections. Fat pockets are easily identifiable as large holes. Numerous macrophages are visible in male and female WT and male, but not female, ASIC2^-/-^/βENaC^m/m^ mouse sections (**Figures 7A, B**). An enlarged image of female WT and ASIC2^-/-^/βENaC^m/m^ samples is shown (**Figure 7C**). Quantitative immunolabeling indicates a profound reduction in the number of liver macrophages (**Figure 7D**).

### 3.8 Plasma lipid profile is improved in ASIC2^-/-^/βENaC^m/m^ mice on HFD

**Figure 8:**
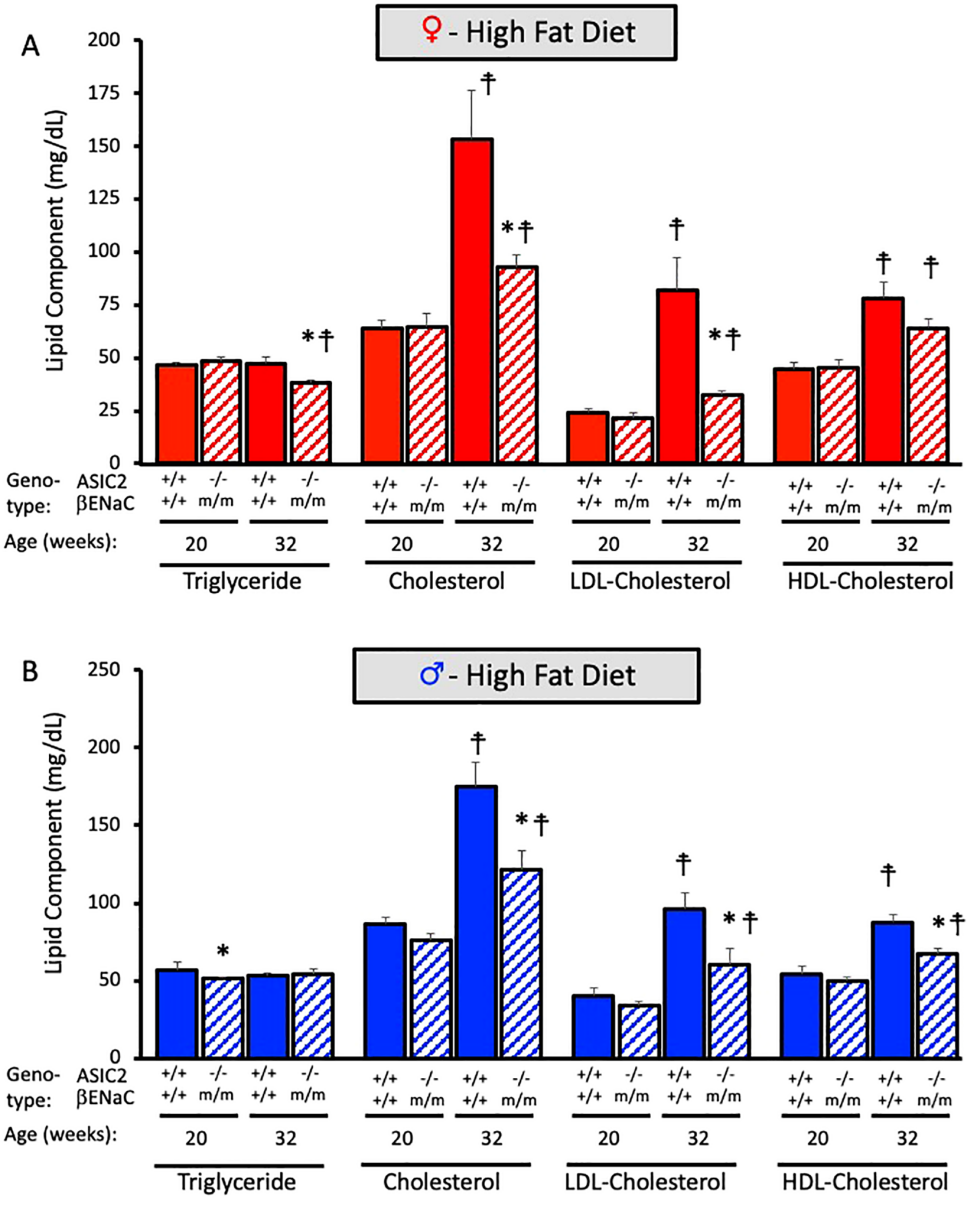
Plasma lipid profile is improved in ASIC2^-/-^/βENaC^m/m^ fed a high-fat diet (HFD) for 20 weeks. **(A)** Triglyceride, cholesterol, LDL, and HDL cholesterols are in HFD female ASIC2^-/-^/βENaC^m/m^ and wild-type (WT) mice. **(B)** Triglyceride, cholesterol, LDL, and HDL cholesterols are in HFD male ASIC2^-/-^/βENaC^m/m^ and WT mice. Red columns represent female individuals; blue columns represent male individuals. Solid columns represent the WT group, and hatched columns represent the ASIC2^-/-^/βENaC^m/m^ group. Statistical analysis included dependent, two-tailed *t*-tests with the Bonferroni correction. Sample sizes *n* = 8–9. The asterisk denotes being significantly different from WT control at a respective age, *p* < 0.025. Modified cross denote being significantly different within genotype at 20 weeks, < 0.025.

We measured plasma triglycerides, cholesterol, LDL cholesterol, and HDL cholesterol and their metabolites before (20 weeks of age) and after (32 weeks of age) HFD. The effect of HFD on the main lipid components in ASIC2^-/-^/βENaC^m/m^ are shown in **Figure 8**. Baseline measurements at 20 weeks indicate that plasma triglyceride, cholesterol, and LDL- and HDL-cholesterol levels are identical between WT and ASIC2^-/-^/βENaC^m/m^ mice (**Figures 8A, B**). In contrast, ASIC2^-/-^/βENaC^m/m^ mice were protected from the 12-week HFD-induced increases in total cholesterol, LDL cholesterol, and HDL cholesterol (**Figures 8A, B**).

## 4 Discussion

Obesity prevalence is continuing to rise globally, and with this comes a need to understand the pathophysiological mechanisms of obesity-related metabolic disease. Genetically modified mouse models have been used for decades to better understand metabolic disease progression. Degenerin proteins are expressed in multiple cell types involved in the pathology of metabolic syndrome, including but not limited to hypothalamic neurons, peripheral neurons, renal and colon epithelia, and endothelial, smooth muscle, and immune cells (4–11, 21, 30, 41, 46–48). In these cell types, degenerins contribute to the neural regulation of autonomic nerve activity, extracellular fluid homeostasis, and blood pressure control (6, 23, 36, 37). To determine if degenerins play an important role in cardiovascular function, we questioned whether mice lacking ASIC2^-/-^/βENaC^m/m^ might have an exaggerated phenotypic response to a high-fat diet. We chose this model for our inaugural study because ASIC2 and βENaC are expressed in many of the cell types listed above and participate in the regulation of blood pressure. Additionally, we rationalized that using a model lacking more than one could exacerbate any phenotype from the loss of the important protein family. Our study provides a novel, yet unexpected, finding: loss of ASIC2 and βENaC leads to remarkable protection against HFD-induced weight gain, intrabdominal fat accumulation, hepatic steatosis, insulin resistance, and plasma lipid profile changes.

Interestingly, we found that loss of ASIC2/βENaC afforded greater protection in female mice. ASIC2^-/-^/βENaC^m/m^ female mice have better glucose tolerance test performance, almost no increase in plasma insulin levels, and minimal fat and macrophage accumulation in the liver in response to chronic HFD. Our findings are consistent with the role of estrogen acting as a preventative factor against metabolic syndrome in our control animals (49, 50). However, whether estrogen enhances the protective response in our ASIC2^-/-^/βENaC^m/m^ mice is unclear.

The most impressive finding in the present study is reduced fat storage in the livers of ASIC2^-/-^/βENaC^m/m^ mice. ECHO MRI assessment indicates 50%–90% less fat content in male and female mice, respectively. The results of the Oil Red O staining demonstrate almost no hepatic lipid accumulation in female ASIC2^-/-^/βENaC^m/m^ mice after 12 weeks of HFD, while significant lipid accumulation is present in the livers of WT female mice. In male mice, the area of Oil Red O staining is similar in male WT and ASIC2^-/-^/βENaC^m/m^ mice; however, the intensity of Oil R O staining is reduced in male ASIC2^-/-^/βENaC^m/m^ mice compared to WT mice (**Figure 6H**, lower right panel) consistent with a lower fat content.

There are several potential mechanisms which could account for this dramatic decrease in hepatic lipid accumulation observed in ASIC2^-/-^/βENaC^m/m^ female and male mice, including increases in hepatic fatty acid oxidation and decreases in hepatic fatty acid uptake. It is possible that alterations in fat absorption from the gastrointestinal system could result in lower levels of hepatic lipid accumulation as well as lower plasma levels of triglycerides and cholesterol. Dietary fat is absorbed through the small intestine and then passes through the liver where it is stored and/or metabolized. While βENaC and ASIC2 messages are not abundantly expressed in healthy human small colon or liver tissue under normal conditions (34, 35), there are several lines of evidence that show that inflammatory pathology may alter the expression of ASIC molecules. First, ASIC expression in certain cells is upregulated by inflammation, which HFDs evoke (17, 46, 48, 51, 52). Second, at least ASIC1a transcript and protein are expressed in rat liver and cultured primary hepatic stellate cells and contribute to the activation of hepatic stellate cells (39, 53). Third, ASIC2 is undetectable in normal hepatocytes but is expressed in a population of injured liver hepatocytes and hepatic stellate cells (34). Hepatic stellate cells are critical mediators of HFD-induced liver fibrosis and injury (38). The importance of ASIC2 in hepatic cell activation has not been addressed and may represent a potential mechanism contributing to HFD-induced liver inflammation. ASIC2 is also expressed in some macrophages and dendritic cell preparations and may contribute to the hepatic inflammatory response (8, 10). Previous studies have shown that loss of peroxisome proliferator-activated receptor alpha (PPARα) in the liver results in inflammation and hyperlipidemia in response to HFD (44, 45), and PPARα agonists are approved for the treatment of dyslipidemia (54). It is possible that PPARα may inhibit the activity of a degenerin channel, possibly ASIC2 or a hybrid ENaC–ASIC channel, that has yet to be identified in hepatic cells, and disruption of this regulatory system in the liver may be a driver for metabolic syndrome development (55). It is unclear how gastrointestinal ENaC may contribute to the metabolic syndrome phenotype; however, ENaC is regulated by insulin (6, 37) and thus may contribute to intracellular signaling affecting glucose utilization within the gut.

The CNS signaling pathway of feeding–satiety–energy expenditure could also play a role in protecting ASIC2^-/-^/βENaC^m/m^ mice from HFD-induced metabolic syndrome. ASIC2 is widely expressed in the brain, including the hypothalamus (20, 40, 41). Data available from the HypoMap single cell expression atlas concentrates ASIC2 expression in the ventromedial hypothalamus and arcuate nucleus, two areas important in metabolic homeostasis (18–20). βENaC is weakly expressed in a disperse population of these neurons (20). Within the arcuate nucleus, both POMC and AgRP neurons express ASIC2 (20). POMC neurons promote energy expenditure and suppress the drive for food consumption, while AgRP neurons function in an opposite manner (18, 20). Whether ASIC2 or βENaC contribute to feeding and/or motor activity is unclear. A recent publication suggests that distance traveled, as a measure of motor activity, is unchanged in ASIC2^-/-^ mice (56). The importance of βENaC in motor activity, energy expenditure, and hunger–satiety has not been studied. Future studies will focus on determining the contribution of the single genotypes (ASIC2^-/-^ or βENaC^m/m^) to the phenotype and will guide our examination of the importance of the central pathways in feeding–satiety–energy expenditure and the small intestine–liver–immune axis.

## 5 Conclusion

Our findings indicate that the degenerin proteins ASIC2 and βENaC are involved in the progression of metabolic syndrome. Animals lacking normal levels of ASIC2 and βENaC display reduced HFD-induced body mass gain, fasting blood glucose, plasma insulin and leptin profiles, liver mass and fat content, macrophage localization to the liver, and pathological circulating lipid profile. Additionally, loss of ASIC2 and βENaC confers a greater protection against the development of insulin resistance and hepatic steatosis in female mice as compared to male mice. The specific underlying mechanisms that contribute to this protection are unclear but will be the focus of future studies. It is possible that agents blocking these signaling pathways could be novel candidates for the treatment of metabolic disease in humans.

## Data Availability

The data are available via figshare doi: 10.6048/m9.figshare.26042863.
